# Novel transport properties of the α-T_3_ lattice with uniform electric and magnetic fields

**DOI:** 10.1038/s41598-022-17288-8

**Published:** 2022-07-29

**Authors:** Fu Li, Qingtian Zhang, Kwok Sum Chan

**Affiliations:** 1 School of Materials and Energy, Guangdong University of Technology, Guangzhou, 510006 Guangdong People’s Republic of China; 2 Guangdong Provincial Key Laboratory of Information Photonics Technology, Guangdong University of Technology, Guangzhou, 510006 Guangdong People’s Republic of China; 3 Department of Physics, City University of Hong Kong, Tat Chee Avenue, Kowloon, Hong Kong People’s Republic of China

**Keywords:** Electronic properties and materials, Electronic devices, Quantum simulation

## Abstract

We report a theoretical study of electronic transport properties of *α*-T_3_ lattice nanoribbons in the presence of uniform electric and magnetic fields. Landau levels with an unexcepted fashion are obtained in the system, and unique flat bands are observed due to the crossed electric and magnetic fields. We found that the nondispersive flat band of *α*-T_3_ lattice is distorted and split to many dispersive energy levels when electric and magnetic fields are applied. A double constriction structure of *α*-T_3_ lattice is considered to investigate the quantum transport in the flat band, and novel quantum transport properties are obtained, which shows great differences from conventional Dirac electrons. Our results show that the flat bands of *α*-T_3_ lattice can also contribute to the quantum transport properties and play an important role in the development of novel Dirac electron device.

## Introduction

Graphene has a flat hexagonal lattice of carbon atoms forming a monoatomic layer^1^, and the electronic and structural characteristics due to the layer structure have attracted widespread attention from engineers and physicists^2–8^. The interest in graphene has prompted the active and comprehensive research in two-dimensional (2D) Dirac materials^9^. Recently, the dice lattice also becomes a very popular 2D Dirac material in research studies, and its band structure is similar to that of graphene, except for a nondispersive flat band found at zero-energy^10–12^. Besides, there is a new type of 2D Dirac material named *α*-T_3_ lattice, which is an interpolation between the dice lattice and graphene^13,14^. When an atom is added to the center of each hexagon of the honeycomb lattice of graphene, the lattice formed is the *α*-T_3_ lattice^15^. A unit cell of an *α*-T_3_ lattice consists of three atoms, the AB atoms in the honeycomb lattice of graphene and the C atom at the centre of the hexagon. The additional atom C only couples with either A or B atom but not both, and the coupling strength is described by the parameter *α*. The electric properties of the *α*-T_3_ lattice changes with the value of the parameter *α*, and the *α*-T_3_ lattice becomes graphene at α = 0 and changes to the dice lattice at α = 1. It is demonstrated that α-T_3_ lattice can be obtained in Hg_1−x_Cd_x_Te at a critical doping with an intermediate value of the parameter $\alpha { = 1/}\sqrt 3$^16^. The optical α-T_3_ lattice has been predicted theoretically, and the value of the parameter α can be varied in these systems^17,18^. In experimental studies, the dice lattice has already been realized by growing a three-layer structure of SrTiO_3_/SrIrO_3_/SrTiO_3_ along the (111) direction^19,20^.

The special lattice structure of the *α*-T_3_ lattice leads to many novel properties, and as a result, it has been intensively studied recently^21–24^. One of the interesting characteristics is the nondispersive flat band^25^. Owing to the recent discovery of the flat band in twisted bilayer graphene systems^26–28^, there is a strong interest in the study of flat band in other materials and systems. The following are examples in which a flat band is found: Lieb lattice^29^, optical lattice systems^30,31^, and 1 T-TaS2 material^32^, *α*-T_3_ lattice^33^. The nondispersive flat band do not contribute to the electron transport due to the zero group velocity; however, the flat band in *α*-T_3_ lattice leads to many peculiar characteristics in quantum transport^34–38^. Moreover, some attention has been devoted to the investigation of *α*-T_3_ lattice with broken flat band. Wang et al.^39^ study the quantum Hall effect in *α*-T_3_ lattice with staggered potential and disorder, and they found that the staggered lattice potential and disorder can break the flat band so as to break the zero Hall plateau of origin *α*-T_3_ lattice. Previous studies show that no zero-energy minimal conductivity is found in clean *α*-T_3_ lattice^40,41^, however, the zero-energy minimal conductivity can be obtained when staggered potential or disorder is considered.

In this study, we investigate the novel electronic transport properties in *α*-T_3_ lattice under the effect of crossed magnetic and electric fields. Though the electronic properties of α-T3 lattice nanoribbons under an external magnetic field have been studied analytically and numerically^13,42^, the transport properties of α-T3 lattice with both magnetic and electric fields are still interesting and important. We found that the zero-energy nondispersive flat band of *α*-T_3_ lattice is distorted when an in-plane electric field and perpendicular magnetic fields are applied, and the nondispersive zero-energy level is split and broadened to many dispersive energy levels. We show how the Landau levels are affected by the transverse electric field, and how the magnetic field changes the broadened distorted flat band levels. We obtain Landau levels with unexpected fashion, and many unique properties for the zero-energy flat band are presented. We also consider a double constriction structure of *α*-T_3_ lattice in the presence of magnetic field, and it is found that electrons in the broadened zero-energy level have very different transport properties. Our findings reveal rich physics of the flat band in *α*-T_3_ lattice and show that the flat band can also contribute to quantum transport.

## Model and methods

**Figure 1:**
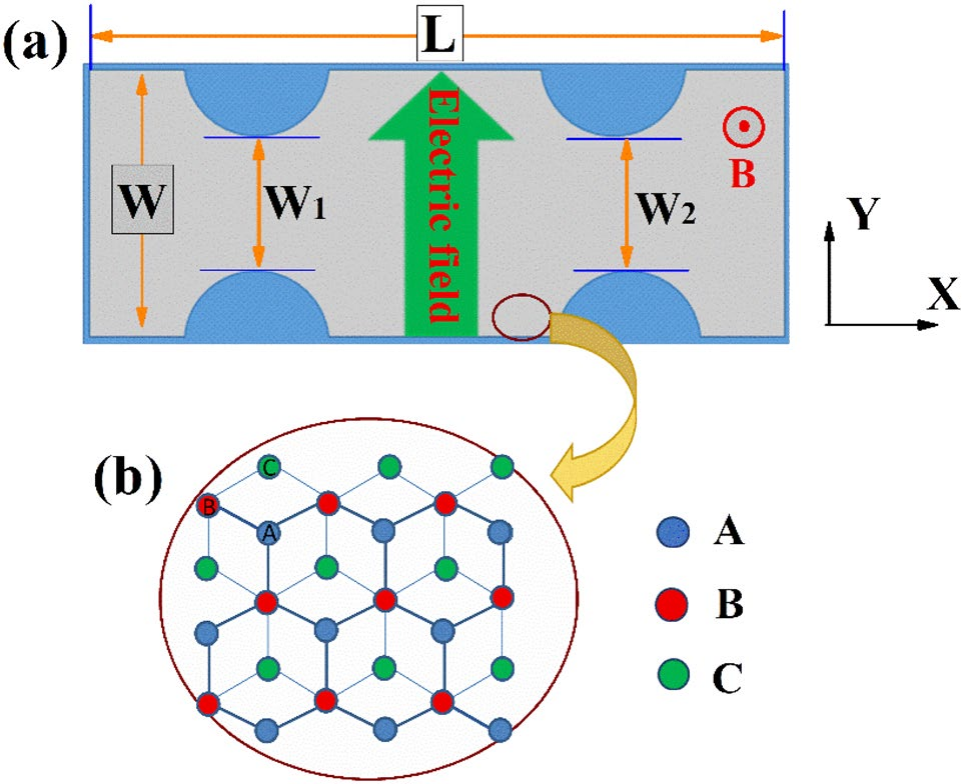
(**a**) Schematic diagram of the proposed device with two nanoscale constrictions, the distances between the two split gates on the left is indicated by W_1_ and W_2_, the width W = 30 nm, the length L = 120 nm. The green arrow represents the direction of the electric field, and the magnetic field is applied perpendicularly to the plane. (**b**) The lattice structure of *α*-T_3_ model. Three atoms in each unit cell are indicated by different colours, ie. A (blue), B atom (red) and C (green).

The schematic diagram of the device structure considered in this study is shown in Fig. 1a. We propose a device with two nanoscale constrictions, and gate electrodes can be fabricated on the top of the *α*-T_3_ lattice to produce the depletion region underneath to create the nanoscale constrictions. The constrictions can also be realized by etching, but it is more convenient to control the size of the constrictions through the gate electrodes. The dimensions of the device used in our numerical calculations are chosen to be L = 120 nm, and W = 30 nm. The sizes of the constrictions are denoted by the parameters W_1_ and W_2_, and they are set to be W_1_ = W_2_ = 15 nm in our study. Here we use a carbon-carbon bond length of 0.142 nm, which is the same as in graphene. The *α*-T_3_ lattice structure is schematically shown in Fig. 1b. It can be seen that each unit cell contains three atoms A (blue), B atom (red) and C (green), and the A and B atoms form a hexagon graphene lattice, while the C atoms are located at the centres of the hexagons. The C atom is only coupled to the B atom of the honeycomb lattice. The hopping amplitude between the A and B atoms is t, and the hopping amplitude between the B and the C atoms is $t^{\prime} = \alpha t$. The parameter *α* plays a very important role in the electronic properties of *α*-T_3_ lattice, and we can choose a value in the interval [0,1] in a theoretical calculation. It is easy to note that we have graphene lattice when *α* = 0, but it is changed to dice lattice when *α* = 1. We can also see in Fig. 1b that we have zigzag and armchair edges along the x and y directions respectively. For a zigzag nanoribbon, we can define a C–A edged nanoribbon if the top atom is the C atom and the bottom is the A atom (see the example in Fig. 1b).

In the tight-binding formulation, the Hamiltonian of an *α*-T_3_ lattice in the presence of a perpendicular magnetic field and an external transverse electric field can be written as

1 $$H = \sum\limits_{\gamma } {(\varepsilon_{\gamma } } + eE_{y} y)c_{\gamma }^{ + } c_{\gamma } + \sum\limits_{{\left\langle {ij} \right\rangle }} {te^{{i\phi_{ij} }} c_{i}^{ + } c_{j} + \sum\limits_{{\left\langle {jk} \right\rangle }} {t^{\prime}e^{{i\phi_{jk} }} c_{j}^{ + } c_{k} } }$$

where $$c_{i,j,k} (c_{i,j,k}^{ + } )$$ are annihilation (creation) operators of electrons on the A B and C sites, which are denoted by $$\gamma = i,j,k$$ indices. The first term is the onsite energy $$\varepsilon_{\gamma }$$ and the external potential $$eE_{y} y$$ induced by the transverse electric field. The second term is the electron hopping between the A and B sites, while the third term is the hopping between the B and C sites. The hopping energy between the A and B sites is t, and the hopping energy between B and C is $$t^{\prime} = \alpha t$$, where $$\alpha$$ is the intermediate parameter of *α*-T_3_ lattice. The summation of $$\left\langle {ij} \right\rangle$$ and $$\left\langle {jk} \right\rangle$$ runs over the nearest neighbor sites, which correspond to the hoppings between the A and B sites and between the B and C sites respectively. The hopping between the A and B sites is prohibited. Owing to a perpendicular magnetic field, a Peierls’ phase $$\phi _{{ij(jk)}} = (2\pi /\phi _{0} )\int_{{j(k)}}^{{i(j)}} {\overset{\lower0.5em\hbox{$\smash{\scriptscriptstyle\rightharpoonup}$}} {A} \cdot d\overset{\lower0.5em\hbox{$\smash{\scriptscriptstyle\rightharpoonup}$}} {l} }$$ is added to the hopping element, where the vector potential is $$\overset{\lower0.5em\hbox{$\smash{\scriptscriptstyle\rightharpoonup}$}} {A} = ( - By,0,0)$$ and the quantum of magnetic flux is $$\phi_{0} = h/e$$. The strength of the magnetic field can be given by the magnetic flux per honeycomb $$\phi = (3\sqrt 3 /2)a^{2} B/\phi_{0}$$**,** and we choose $$\phi { = }0.0015$$ in our numerical calculations. The intermediate parameter is set to be α = 0.5 in all the numerical calculations. In our numerical calculations, t = 3 eV and a = 1.42 Å are used, which is convenient to compare the results with graphene. All numerical calculations were performed using the Kwant tight-binding code^43^.

## Result and discussion

**Figure 2:**
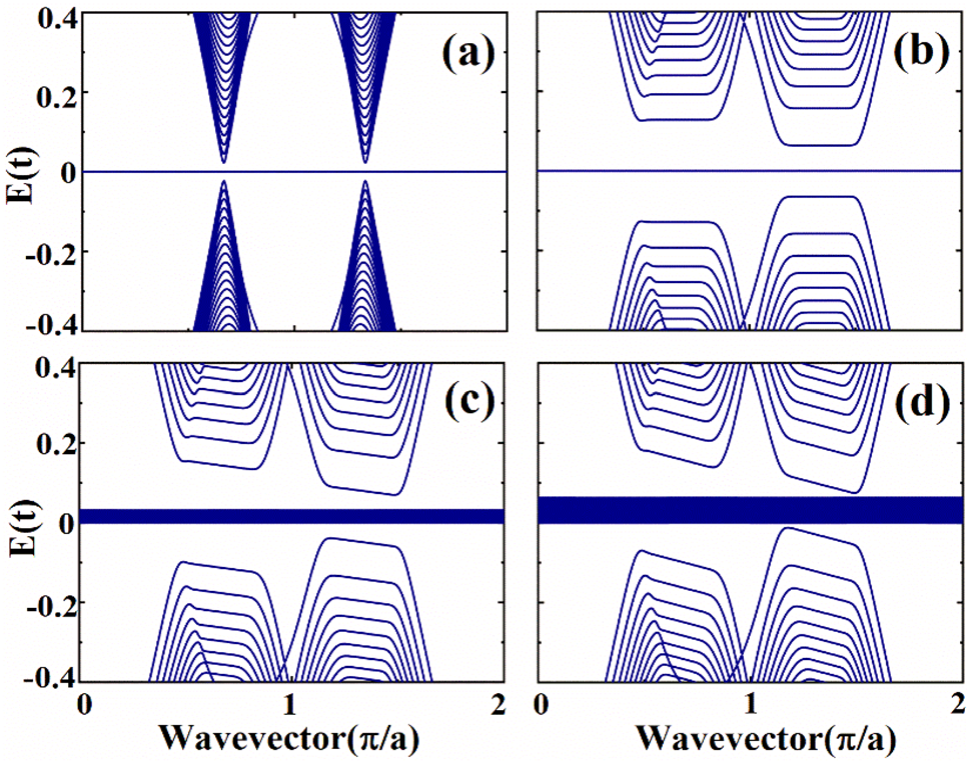
Band structures of zigzag ribbons with various magnetic flux $\phi$ and electric field $eE_{y}$. (**a**) $\phi \;{ = }\;0$ and $eE_{y} = 0$. (**b**) $\phi \;{ = }\;0.0015$ and $eE_{y} = 0$. (**c**) $\phi \;{ = }\;0.0015$ and $eE_{y} = 0.001t{\text{/nm}}$. (**d**) $\phi \;{ = }\;0.0015$ and $eE_{y} = 0.002\;t{\text{/nm}}$.

In order to study the quantum transport properties of *α*-T_3_ lattice under the effect of a magnetic field and electric field, we present the band structures of zigzag ribbons with parameters *α* = 0.5 for various magnetic and electric fields. In Fig. 2a, the band structure for *α*-T_3_ lattice with $\phi \;{ = }\;0$ and $eE_{y} = 0$ is shown, and it is noted that we obtain a zero-energy nondispersive flat band, which is a very interesting property of the *α*-T_3_ lattice. We have two valleys shown in the band structure, and the subbands in the two valleys are the same. In Fig. 2b, the band structure for *α*-T_3_ lattice ribbon with $\phi \;{ = }\;0.0015$ and $eE_{y} = 0$ is presented, and we can see that flat energy bands are formed, which indicates the formation of Landau levels. Moreover, the magnetic field enlarges the energy band gap between conduction and valence bands, and the subbands in the two valleys are different. The magnetic field can induce valley polarization in *α*-T_3_ lattice ribbon, which is different from the graphene nanoribbon. In Fig. 2c, we consider a perpendicular magnetic field and a transverse electric field, and the values for the parameters are set to be $\phi \;{ = }\;0.0015$ and $eE_{y} = 0.001t{\text{/nm}}$. Comparing the Landau levels shown in Fig. 2b with Fig. 2c we can note that the transverse electric field modify the Landau levels in an unexpected fashion. It is found that the zero-energy nondispersive flat band is broadened to many dispersive energy levels. As we know that the group velocity of electrons in a perfect flat band shown in Fig. 2a,b is zero, and the perfect flat bands will not contribute to quantum transport. The broadened flat band levels are not perfect flat band anymore, and they are distorted by the transverse electric field, which will be further discussed below. According to previous studies^39,44^, a stagger potential bends the flat band. We believe that the applied transverse electric field also bends the band by creating potential differences between the A, B and C atoms in *α*-T_3_ lattice, an effect similar to what happens under a stagger potential. In Fig. 2d, we also consider both electric and magnetic fields in the ribbon, but we consider a stronger electric field. It is noted that the energy range of the broadened flat band levels is enlarged when a stronger electric field is considered. The transverse electric field not only modifies the Landau levels in an unexpected fashion, but also changes the zero-energy nondispersive flat band to distorted flat bands, which are dispersive. We believe that the transverse electric field plays a very important role in the transport properties of *α*-T_3_ lattice by changing the flat band of *α*-T_3_ lattice.

We also considered zigzag ribbon with B–B edge and armchair ribbon. As in C–A edge zigzag ribbon, there is a zero energy non-dispersive flat band, which is split under magnetic and electric fields into distorted flat bands, which are dispersive. We expect the B–B edge zigzag ribbons and armchair ribbons will have similar transport characteristics of the C–A edge zigzag ribbons. Owing to space limitation, we do not show the figures here.

**Figure 3:**
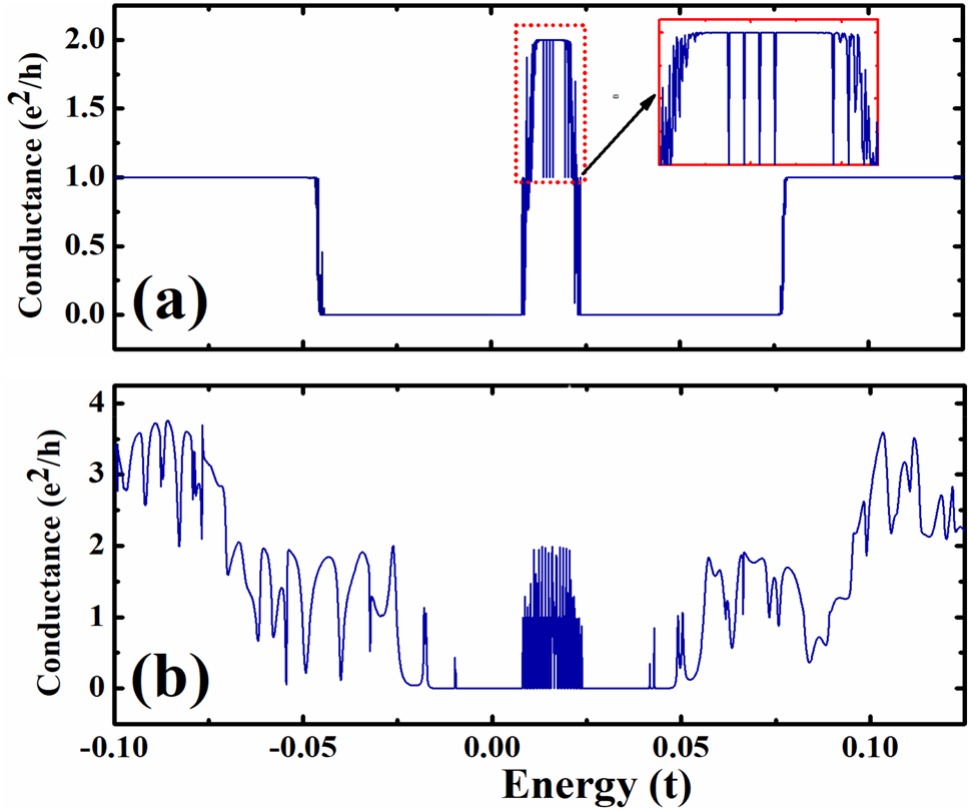
Conductance of the device with two nanoscale constrictions plotted as a function of energy. (**a**) Both magnetic and electric fields are considered in the device with the parameters $\phi \;{ = }\;0.0015$ and $eE_{y} = 0.001t{\text{/nm}}$. (**b**) Only electric field is considered with the parameters $\phi \;{ = }\;0$ and $eE_{y} = 0.001t{\text{/nm}}$.

**Figure 4:**
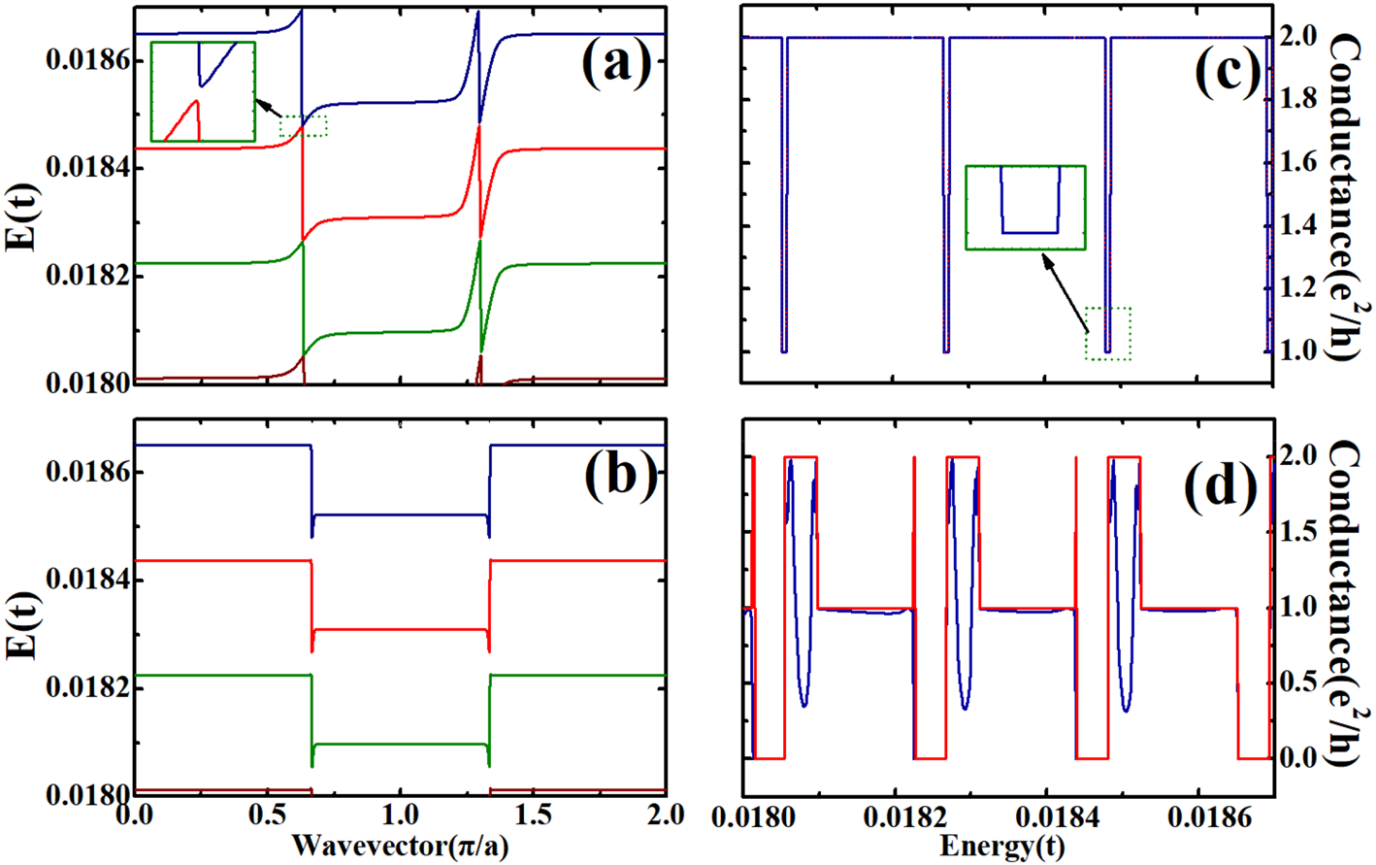
Band structures and conductance for the device in a small energy region. (**a**) Band structure for zigzag ribbon with both magnetic and electric fields. (**b**) Band structure for zigzag ribbon with only electric field. (**c**) Conductance for the device with and without nanoscale constrictions while both magnetic and electric fields are considered. (**d**) Conductance for the device with and without nanoscale constrictions while only electric field is considered.

In Fig. 3, we plotted the conductance of our proposed device as a function of energy, and we compare the conductances of the device with and without magnetic field. The results with both magnetic and electric fields are shown in Fig. 3a, and the case with only the electric field is shown in Fig. 3b. For the case with both magnetic and electric fields $\phi \;{ = }\;0.0015$ and $eE_{y} = \;0.001t{\text{/nm}}$, we found that the conductance shows perfect plateaus. When ${\text{E}} < - 0.0{\text{45t}}$ or ${\text{E}}\;{ > }\;0.076{\text{t}}$, the conductance shows perfect conductance plateaus with ${\text{G}}\;{ = }\;{{e^{2} } \mathord{\left/ {\vphantom {{e^{2} } h}} \right. \kern-\nulldelimiterspace} h}$, which is a typical characteristic of magnetic field. It is interesting to note that the conductance in the range $0.00{8}t < E < 0.0{25}t$ also has some conductance plateaus, but there are sudden changes of the conductance in this region. To understand the transport properties in the energy range with flat bands, we will focus on the band structure and conductance in this range, which will be shown in Fig. 4. In Fig. 3b, it can be noted that the conductance for the device with a non-zero electric field and zero magnetic field oscillates significantly, and this is caused by the scatterings induced by the two nanoscale constrictions of the system. The transverse electric field caused distortion of the flat band levels and spread them in an energy range, and this results in a nonzero conductance in the range 0.008*t* < *E* < 0.025*t*. We can see that the conductance in this range also oscillates with the energy.

As shown in Fig. 3, the conductance of the device shows some interesting oscillating characteritsics in the energy range of the broadened and distorted flat band. In order to understand these interesting characteristics, we need to look at the features of the band structure and conductance in a smaller energy range. In Fig. 4, we show the band structure and conductance in the small energy range of 0.018t–0.0187t. We also plot the conductance of the device without the two nanoscale constrictions in Fig. 4c,d for comparing the effects of with and without the constrictions. In Fig. 4c, where both electric field and magnetic field are considered, we can see that the conductances for the devices with and without constrictions are the same, and the two lines are actually overlapped. When there is a magnetic field, no scattering effect is found for the distorted flat bands, so the conductance is either a ${{e^{2} } \mathord{\left/ {\vphantom {{e^{2} } h}} \right. \kern-\nulldelimiterspace} h}$ or $2{{e^{2} } \mathord{\left/ {\vphantom {{e^{2} } h}} \right. \kern-\nulldelimiterspace} h}$ plateau. The two constrictions cannot affect the transport properties. When the energy changes, the conductance can be switched between ${{e^{2} } \mathord{\left/ {\vphantom {{e^{2} } h}} \right. \kern-\nulldelimiterspace} h}$ and $2{{e^{2} } \mathord{\left/ {\vphantom {{e^{2} } h}} \right. \kern-\nulldelimiterspace} h}$, although the conductance plateau of ${{e^{2} } \mathord{\left/ {\vphantom {{e^{2} } h}} \right. \kern-\nulldelimiterspace} h}$ is very narrow in energy in comparison with the plateau of $2{{e^{2} } \mathord{\left/ {\vphantom {{e^{2} } h}} \right. \kern-\nulldelimiterspace} h}$. This can be understood in terms of the features of the distorted flat bands shown in Fig. 4a. Under both the electric field and magnetic field, there are degenerate energy states with different wavevectors. For example, in the energy range between 0.01853 and 0.0186t, you can find four energy states with the same energy but with different wavevectors. Among the four states, two have positive velocities (positive slopes) and two have negative velocities. Each of the two states with positive velocities can contribute a conductance of ${{e^{2} } \mathord{\left/ {\vphantom {{e^{2} } h}} \right. \kern-\nulldelimiterspace} h}$ and thus the total conductance is $2{{e^{2} } \mathord{\left/ {\vphantom {{e^{2} } h}} \right. \kern-\nulldelimiterspace} h}$.

In Fig. 4d, we show the conductance for the device with only the electric field, and the blue and red lines correspond to the cases with and without constrictions respectively. It is noted that the constrictions change the transport properties of the device, and the conductance is no longer an integral of ${{e^{2} } \mathord{\left/ {\vphantom {{e^{2} } h}} \right. \kern-\nulldelimiterspace} h}$, which is caused by the electron scatterings induced by the constrictions. Without the constrictions, the conductance is quantized into integral of ${{e^{2} } \mathord{\left/ {\vphantom {{e^{2} } h}} \right. \kern-\nulldelimiterspace} h}$. Three possible values of conductance can be found in this energy range in the device without constrictions, 0, ${{e^{2} } \mathord{\left/ {\vphantom {{e^{2} } h}} \right. \kern-\nulldelimiterspace} h}$ and $2{{e^{2} } \mathord{\left/ {\vphantom {{e^{2} } h}} \right. \kern-\nulldelimiterspace} h}$. With the constriction, this quantization effect is destroyed. The quantization effect comes from the distorted flat band, which is shown in Fig. 4b. There are energy ranges in which there are two states with the same energy but with different wave vectors and each of these states can give a conductance of ${{e^{2} } \mathord{\left/ {\vphantom {{e^{2} } h}} \right. \kern-\nulldelimiterspace} h}$. As a result, the total conductance is $2{{e^{2} } \mathord{\left/ {\vphantom {{e^{2} } h}} \right. \kern-\nulldelimiterspace} h}$. For energy range in which only one state is found for an energy, the conductance is ${{e^{2} } \mathord{\left/ {\vphantom {{e^{2} } h}} \right. \kern-\nulldelimiterspace} h}$ .There are gaps between the distorted flat bands and as a result, there are energies at which the conductance is zero.

**Figure 5:**
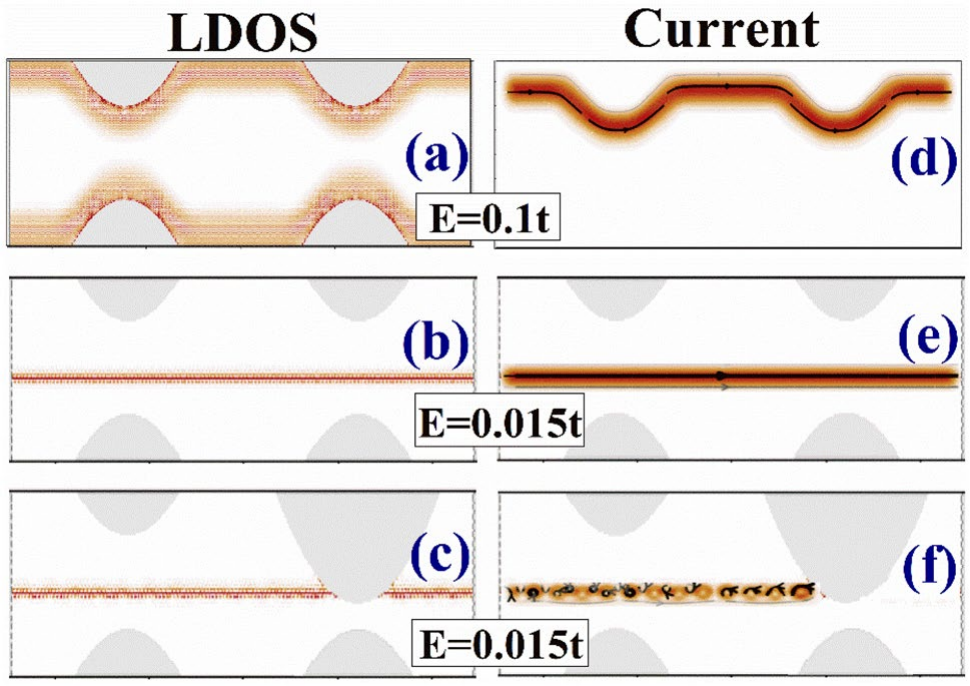
(**a**) and (**d**) show the LDOS and current of the device respectively for E = 0.1t. (**b**) and (**e**) show the LDOS and current of the device respectively for E = 0.015t, and the shape and the size of the device are the same as that in Fig. 1(**a**). (**c**) and (**f**) show the LDOS and current of the device respectively for E = 0.015t, but the sizes of the constrictions are changed to W_1_ = 15 nm and W_2_ = 5 nm. The magnetic flux and electric field are $\phi \;{ = }\;0.0015$ and $eE_{y} = 0.001t{\text{/nm}}$.

To understand the transport properties of the device, we plot the local density of states (LDOS) and currents in Fig. 5. In Fig. 5a,d, the LDOS and current of the device for energy E = 0.1t in the conductance plateau are shown, and we can see that the electrons are transported in the channels confined at the edges. It is noted in Fig. 5d that for E = 0.1t in the conductance plateau, current flows on the top edge when electrons come from the left lead, and no current is found inside the device or on the bottom edge. For the electrons coming from the right, the current only flows on the bottom edge, but we do not present the results in Fig. 5. This means for this energy the current is carried by modes localized at the edges. Next, we consider the transport properties of the broadened distorted flat band levels in Fig. 5b,e, where the LDOS and current for E = 0.015t are shown. We can see from Fig. 5b,e that the current and LDOS for E = 0.015t localized inside the device at a distance from the edges. We also find that the location of the current flow can be changed if we change the energy, which is an interesting feature of the distorted flat band. When E = 0, at the lower edge of the band, the electron state is localized near to the lower edge of the ribbon. When E increases, the state moves towards the upper edge of the ribbon. When E is at the upper edge of the band, the electron state is localized near to the upper edge of the ribbon. Owing to space limitation, we do not show all the figures of the current distribution of these states with different energies. Furthermore, it is natural for us to examine how the current in the distorted flat band is affected by the constrictions. In Fig. 5c,f, the size of the constriction is changed to W_1_ = 15 nm and W_2_ = 5 nm. We can see that the current cannot flow from the left lead to the right lead, when the conduction channel is blocked by the smaller constriction. Which is different from an edge state. A constriction or a defect on the edge do not affect the transport characteristics of an edge state, and no backscattering is obtained. However, the current carried by the distorted flat band levels are totally cut off by the larger constriction shown in Fig. 5c,f.

B–B edge zigzag ribbons and armchair ribbons have similar transport characteristics as the C–A edge zigzag ribbons, such as the localization of the distorted states in the ribbon with location dependent on the energy. These states are also affected by the constriction as in C–A edge zigzag ribbons. Owing to space limitations we do not show the results here.

**Figure 6:**
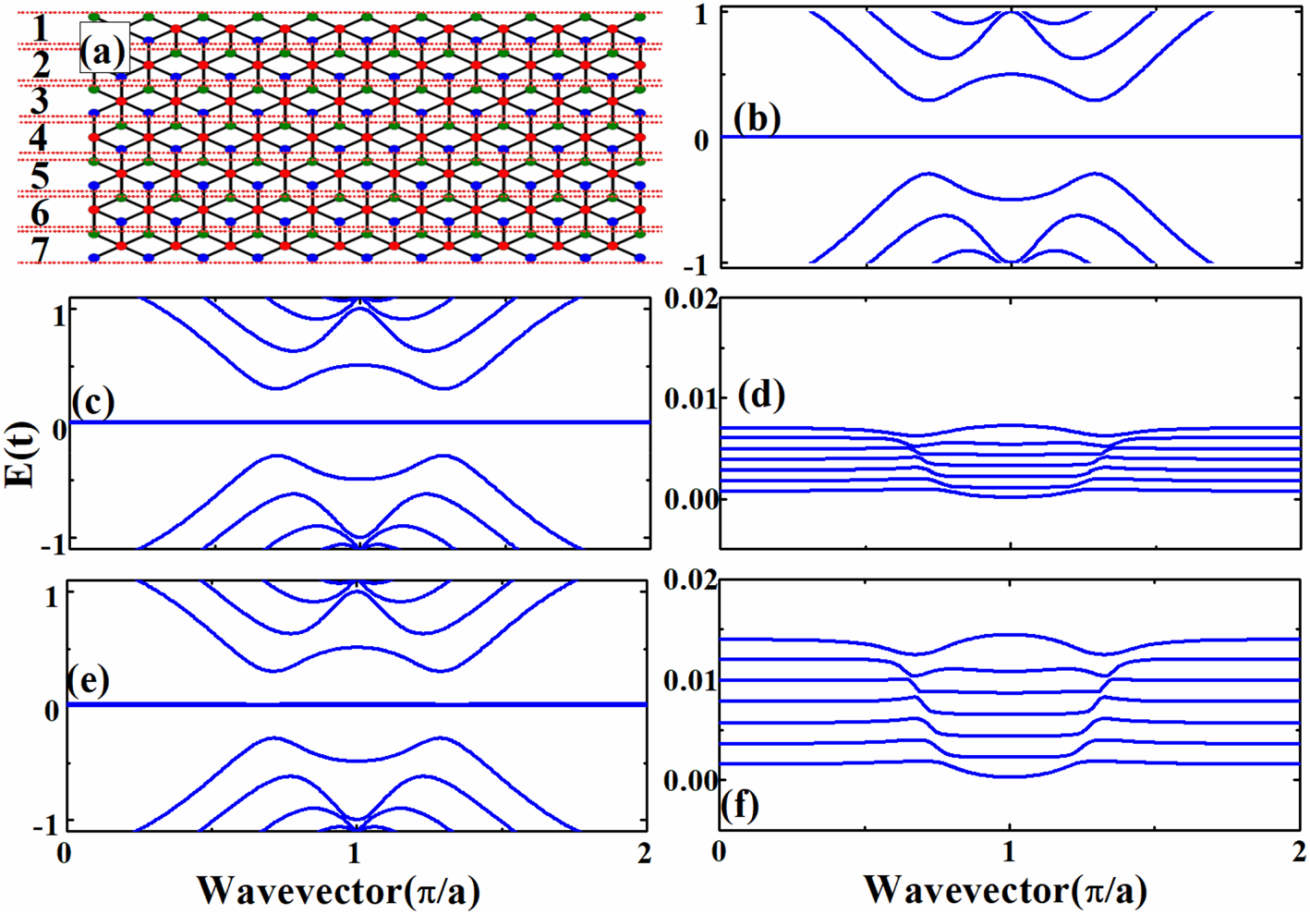
The changes in the flat band of a narrow zigzag *α*-T_3_ lattice nanoribbon with a weaker and a stronger transverse electric field. (**a**) A narrow zigzag *α*-T_3_ lattice nanoribbon. (**b**) Band structure of the narrow *α*-T_3_ lattice ribbon with $eE_{y} = 0$. (**c**) Band structure of the narrow *α*-T_3_ lattice ribbon with $eE_{y} = 0.005t{\text{/nm}}$. (**d**) The enlargement of a part of the flat band of (**c**). (**e**) Band structure of the narrow *α*-T_3_ lattice ribbon with $eE_{y} = 0.01t{\text{/nm}}$. (**f**) The enlargement of a part of the flat band of (**e**).

At last, we consider the effects of transverse electric fields on the flat band. We use a very narrow *α*-T_3_ lattice nanoribbon as an example, and different strength of electric fields are considered. In Fig. 6a, we show the narrow *α*-T_3_ lattice nanoribbon considered. To explain the dependence of the number of the flat band levels on the width of the nanoribbon, we divide the narrow *α*-T_3_ lattice nanoribbon into seven regions, which are marked and labelled in Fig. 6a. In each region, there are two zigzag atom chains. The number of regions is the same as the number of the flat band levels; for example, we obtain seven flat band levels in Fig. 6d,f, because the ribbon under consideration contains seven regions. Figure 6b shows the band structure of the *α*-T_3_ lattice nanoribbon with no magnetic field and no electric field, and we can only see one zero-energy nondispersive flat band. In Fig. 6c, we still consider the band structure of the *α*-T_3_ lattice nanoribbon, but with a transverse electric field $eE_{y} = 0.005t{\text{/nm}}$ applied to the ribbon. It seems that the difference between Fig. 6b,c can be neglected, but significant difference can be found when we enlarge a part of Fig. 6c, which is shown in Fig. 6d. In Fig. 6d, our focus is on the changes of the flat band after we add the transverse electric field. We can see that, the zero-energy degenerate nondispersive flat band is distorted and split to seven dispersive energy levels. When a stronger transverse electric field $eE_{y} = 0.01t{\text{/nm}}$ is applied to the ribbon, and the distorted levels are further split as shown in Fig. 6e,f. Comparing Fig. 6f with Fig. 6d, we note that the separations between the split energy levels increase when the transverse electric field is increased. In Fig. 6d, the energy width of the distorted flat band is $0 < E < 0.007t$, and the energy width of the distorted flat band in Fig. 6f is $0 < E < 0.014t$. It is obvious that in some region of the distorted levels the group velocity is not zero, when there is an applied transverse electric field. This characteristic provides us a way to control the transport properties of an *α*-T_3_ lattice ribbon, which can be used to develop novel electronic devices.

## Conclusions

In summary, we proposed a double constriction structure to study the electronic transport properties of *α*-T_3_ lattice nanoribbons in the presence of a perpendicular magnetic field and a transverse electric field. It is found that we can obtain a nondispersive zero-energy level in the zigzag *α*-T_3_ lattice nanoribbons when no external fields are considered. Landau levels are obtained in zigzag *α*-T_3_ lattice nanoribbons when a perpendicular magnetic field is applied, and a transverse electric field can modify the Landau levels in an unexcepted way. More interestingly, the transverse electric field can distort the nondispersive zero-energy level, and the flat band is broadened to many dispersive energy levels. Though the flat band in *α*-T_3_ lattice make no contribution to the electron transport due to the zero-group velocity, however, the distorted flat band levels show unique transport properties in our proposed double constriction structure. This study reveals the rich physics of *α*-T_3_ lattice with crossed uniform magnetic and electric fields, and it will contribute to the future theoretical and experimental investigations of other dice lattice materials with flat band.

## Data Availability

All data needed to evaluate the conclusions of this study are available in the main text. The data that support the findings of this study are available from the corresponding authors upon reasonable request.
